# Successful Implant Rehabilitation Following Failed Root Canal Treatment Using Ridge Preservation and Delayed Implant Placement: A Case Report

**DOI:** 10.7759/cureus.113097

**Published:** 2026-07-21

**Authors:** Maria I Clavijo, Andrea D Aguana Bruzzo, Keily J Portillo Avila, Ana C Garcia, Erides E Atencio Gonzalez

**Affiliations:** 1 Dentistry, Clavijo Dental, Boynton Beach, USA; 2 Dentistry, Family Dental Associates, Margate, USA; 3 Dentistry, Dental Loft, San Pedro Sula, HND; 4 Dentistry, Private Practice, Key Largo, USA; 5 Dentistry, Lake Park Dental, Tampa, USA

**Keywords:** bone graft, collagen membrane, delayed implant placement, dental implant, endodontic failure, implant-supported crown, osseointegration, ridge preservation, socket preservation, straumann implant

## Abstract

Dental implants have become a predictable treatment option for replacing teeth with an unfavorable prognosis. When extraction of a failed endodontically treated tooth is indicated, preservation of the alveolar ridge is essential to minimize post-extraction bone resorption and facilitate future implant placement.

This case report describes the successful rehabilitation of a mandibular first molar following extraction, ridge preservation, delayed implant placement, and definitive prosthetic restoration using a digital workflow.

A 68-year-old male patient presented with a previously endodontically treated mandibular first molar (tooth #19) exhibiting persistent periapical pathology and an unfavorable long-term prognosis. Following clinical and radiographic evaluation, the tooth was atraumatically extracted. Ridge preservation was performed using deproteinized bovine bone mineral (DBBM) (techBiomat bone®, Technology in Biomaterials S.L., Barcelona, Spain) covered with a resorbable collagen membrane, and primary closure was achieved using 3-0 resorbable catgut sutures. After a three-month healing period, adequate bone regeneration was confirmed clinically and radiographically, allowing placement of a 5.0 × 14 mm Straumann dental implant with a healing abutment. Following an additional three months to allow complete osseointegration and soft tissue maturation, the restorative phase was initiated using a digital intraoral scan with a scan body to fabricate a definitive implant-supported crown. Final clinical and radiographic evaluation demonstrated successful osseointegration, healthy peri-implant soft tissues, appropriate marginal adaptation, stable crestal bone levels, satisfactory occlusion, and favorable esthetic and functional outcomes.

This case highlights the clinical predictability of ridge preservation using DBBM and a resorbable collagen membrane prior to delayed implant placement and demonstrates the successful integration of contemporary digital restorative techniques for implant-supported rehabilitation in an older adult male patient.

## Introduction

Dental implants have become the preferred treatment modality for replacing missing teeth because of their high long-term survival rates, predictable functional outcomes, and favorable esthetic results. Successful implant therapy depends not only on proper implant placement but also on the preservation of adequate alveolar bone volume following tooth extraction. Excessive post-extraction bone remodeling may compromise future implant placement by reducing ridge width and height, making ridge preservation an important component of comprehensive treatment planning [1-4].

Endodontic treatment is highly successful for maintaining natural dentition; however, failures may occur because of persistent microbial infection, inadequate root canal debridement, coronal leakage, procedural complications, vertical root fractures, or persistent periapical pathology. When a tooth presents with an unfavorable prognosis and retreatment is unlikely to achieve long-term success, extraction followed by implant-supported rehabilitation represents a predictable treatment alternative that restores function and esthetics while preventing the progression of chronic infection [5-7].

Following tooth extraction, physiologic remodeling of the alveolar ridge begins immediately and may result in substantial horizontal and vertical bone loss during the first several months of healing. Ridge preservation procedures have therefore become widely accepted to reduce dimensional changes of the alveolar process and facilitate future implant placement. Deproteinized bovine bone mineral (DBBM) is among the most extensively investigated grafting materials because of its excellent biocompatibility, osteoconductive properties, and ability to maintain ridge dimensions during healing. The addition of a resorbable collagen membrane further enhances guided bone regeneration by stabilizing the graft material and excluding soft tissue cells from the healing socket, thereby promoting more favorable bone regeneration [8-12].

Delayed implant placement following ridge preservation has demonstrated predictable clinical outcomes with high survival rates when adequate healing and osseointegration are achieved. Advances in digital dentistry have further improved implant rehabilitation through the use of intraoral scanning, scan bodies, and computer-aided design/computer-aided manufacturing (CAD/CAM) technology, allowing precise fabrication of implant-supported restorations with improved efficiency and accuracy [13-20].

Clinical decision making following endodontic treatment failure requires careful evaluation of the tooth's restorability, the extent of periapical disease, periodontal status, remaining tooth structure, patient-related factors, and the predictability of retreatment. While nonsurgical retreatment or endodontic surgery may allow preservation of the natural dentition in selected cases, extraction followed by implant-supported rehabilitation may represent the most predictable long-term treatment option when the prognosis is unfavorable. In such situations, preservation of the alveolar ridge becomes particularly important to minimize post-extraction dimensional changes that could compromise future implant placement. Although the effectiveness of ridge preservation has been widely documented, reports illustrating its integration with delayed implant placement and a fully digital restorative workflow remain valuable because they demonstrate the clinical decision-making process and the sequential management of patients requiring implant rehabilitation after failed endodontic therapy.

The present case report describes the comprehensive management of a failed endodontically treated mandibular first molar in a 68-year-old male patient through atraumatic extraction, ridge preservation using DBBM and a resorbable collagen membrane, delayed placement of a 5.0 × 14 mm Straumann dental implant, and definitive implant-supported rehabilitation using a fully digital restorative workflow.

## Case presentation

A 68-year-old patient presented for evaluation of a previously endodontically treated mandibular left first molar (tooth #19) with persistent symptoms and concern regarding the long-term prognosis of the tooth. Clinical and radiographic examination revealed failure of the previous root canal treatment associated with persistent periapical pathology, indicating an unfavorable prognosis for long-term retention (Figure 1).

**Figure 1:**
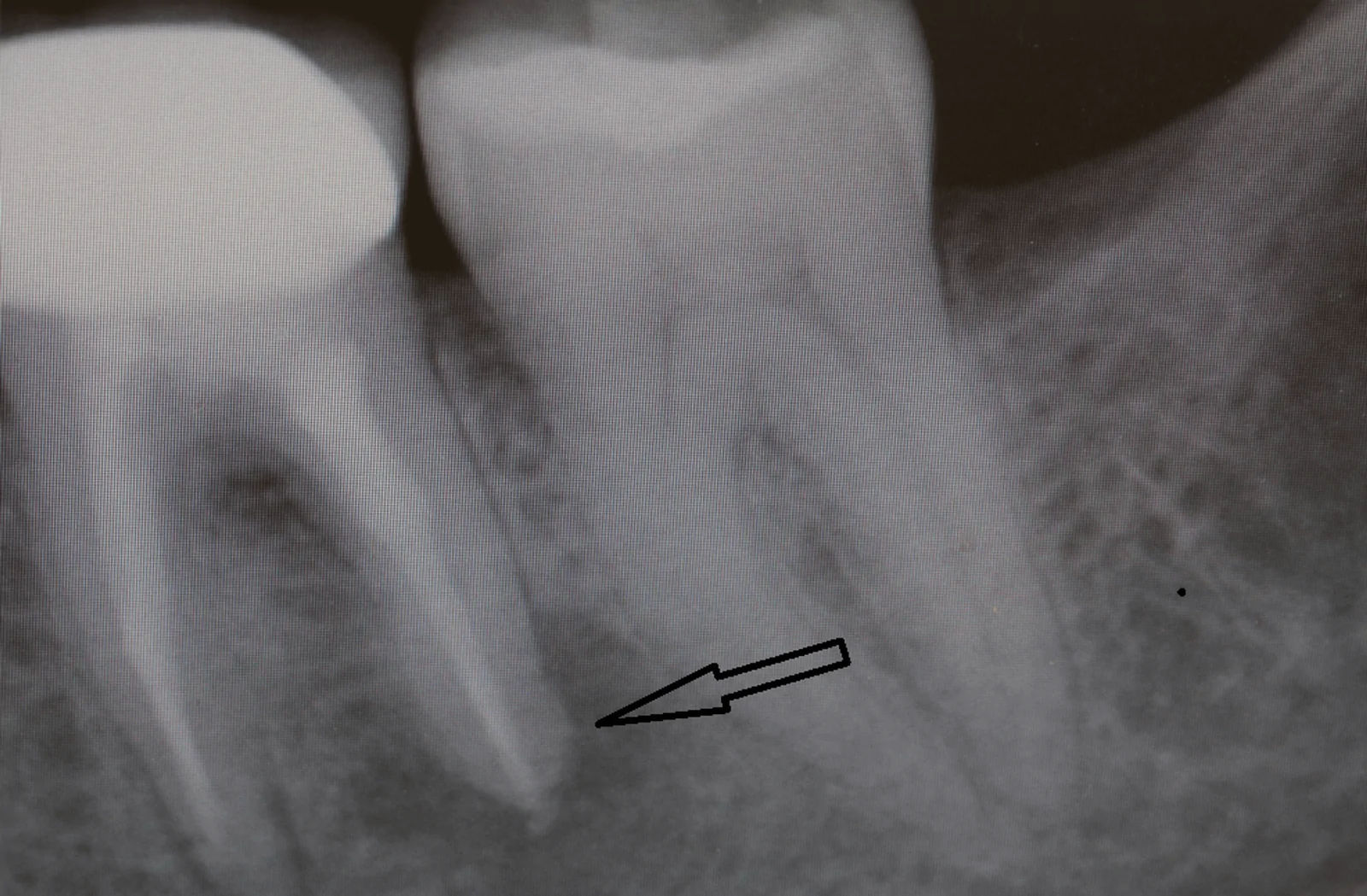
Preoperative periapical radiograph. Preoperative periapical radiograph of the mandibular left first molar (tooth #19). The black arrow indicates the persistent periapical radiolucency surrounding the root apices, consistent with endodontic treatment failure and an unfavorable long-term prognosis.

The patient's medical history was reviewed and revealed no systemic conditions or medications that would contraindicate oral surgical or implant treatment. The patient was classified as American Society of Anesthesiologists (ASA) Physical Status I. His dental history was significant for previous endodontic treatment of tooth #19.

Treatment options, including nonsurgical endodontic retreatment, endodontic surgery, and extraction followed by implant-supported rehabilitation, were discussed with the patient. After reviewing the risks, benefits, and expected outcomes of each option, the patient elected to undergo extraction followed by delayed implant rehabilitation.

The procedure was performed under local anesthesia. Tooth #19 was atraumatically extracted while preserving the surrounding alveolar bone. The extraction socket was thoroughly curetted and irrigated to remove all granulation tissue and residual inflammatory tissue. The extraction socket following debridement demonstrated preservation of the surrounding alveolar socket walls prior to ridge preservation (Figure 2).

**Figure 2:**
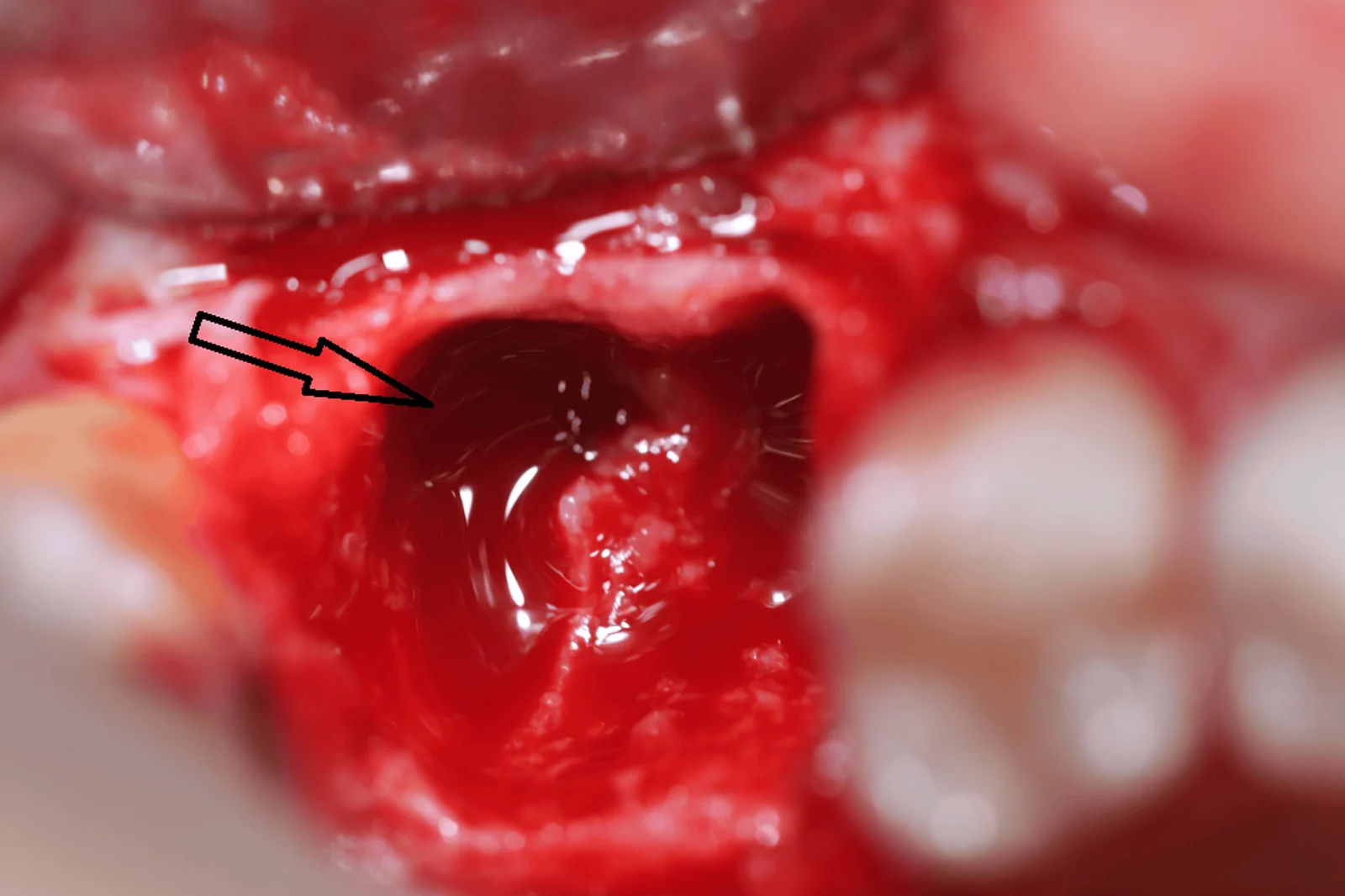
Intraoperative view following atraumatic extraction. Intraoperative clinical photograph obtained immediately after atraumatic extraction of the mandibular left first molar (tooth #19). The black arrow indicates the extraction socket following thorough debridement and irrigation, demonstrating preservation of the surrounding alveolar socket walls prior to ridge preservation using deproteinized bovine bone mineral (DBBM) (techBiomat bone®, Technology in Biomaterials S.L., Barcelona, Spain) and a resorbable collagen membrane.

Ridge preservation was then performed using DBBM (techBiomat bone®, Technology in Biomaterials S.L., Barcelona, Spain), which was placed into the extraction socket to preserve alveolar ridge dimensions and minimize post extraction bone resorption. The grafted socket was covered with a resorbable collagen membrane to stabilize the graft material and promote guided bone regeneration. Primary wound closure was achieved using 3-0 resorbable catgut sutures (Figure 3).

**Figure 3:**
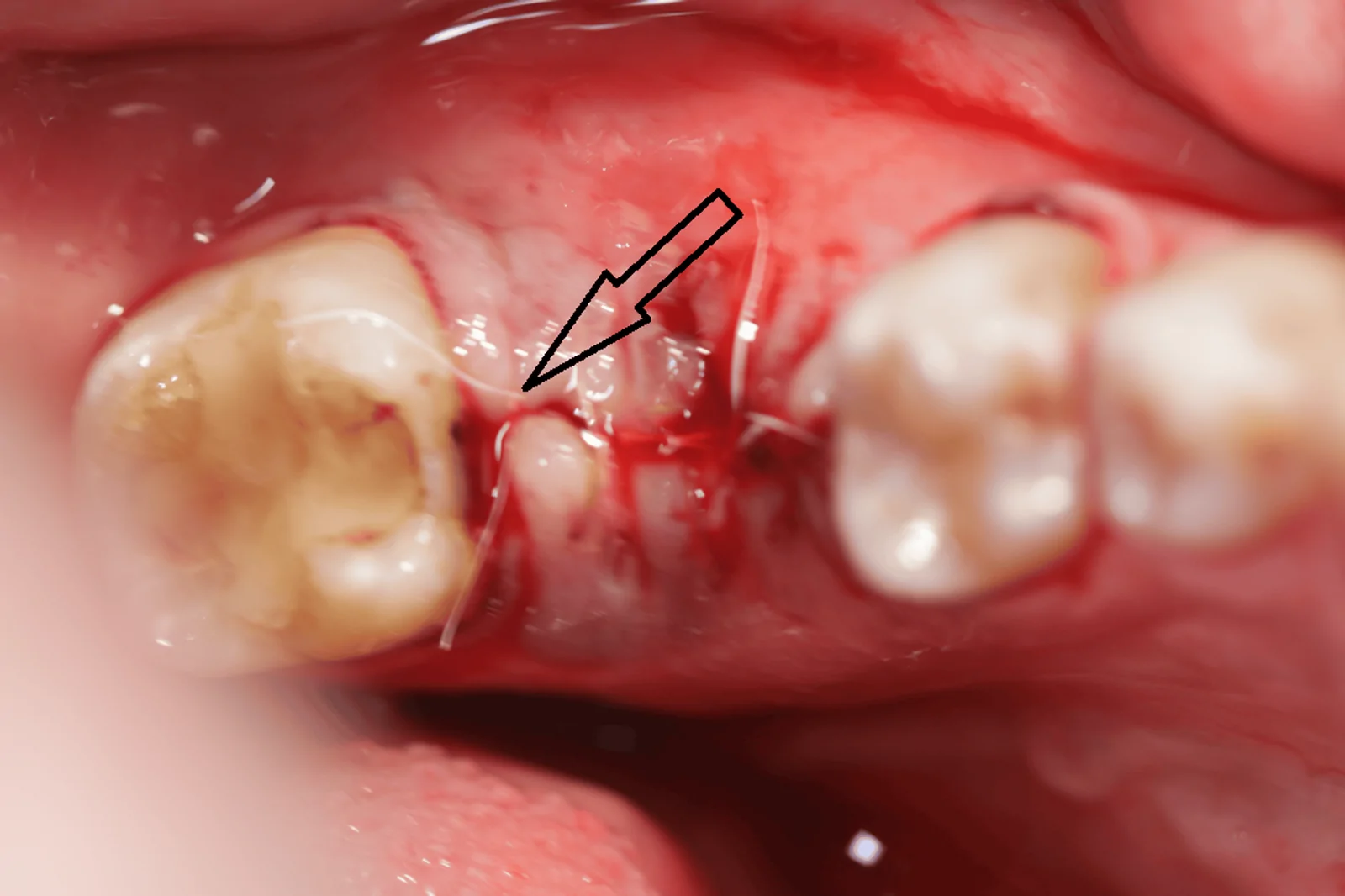
Primary closure following ridge preservation. Intraoperative clinical photograph demonstrating primary closure of the extraction socket following ridge preservation using deproteinized bovine bone mineral (DBBM) (techBiomat bone®, Technology in Biomaterials S.L., Barcelona, Spain) and a resorbable collagen membrane. The black arrow indicates the grafted extraction socket following placement of the regenerative materials and stabilization with 3-0 resorbable catgut sutures to achieve primary wound closure. The wound margins are well approximated, facilitating uneventful healing.

Radiographic evaluation performed three months after ridge preservation demonstrated approximately 12 mm of radiographic bone fill within the preserved extraction socket, with preservation of the alveolar ridge sufficient to permit placement of a 5.0 × 14 mm Straumann dental implant without the need for additional bone augmentation (Figure 4).

**Figure 4:**
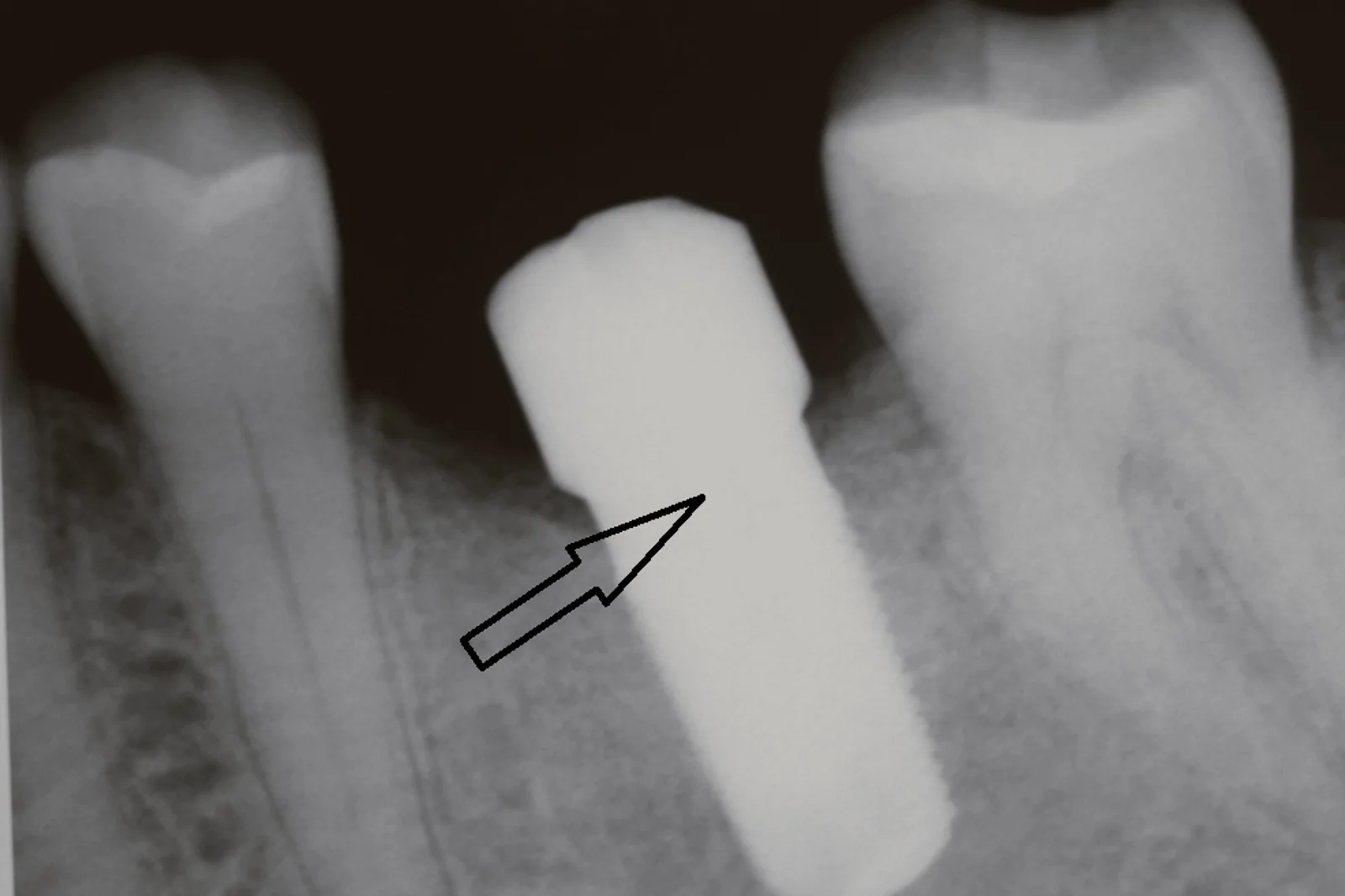
Implant placement after ridge preservation. Periapical radiograph obtained three months after ridge preservation, demonstrating placement of a 5.0 × 14 mm Straumann dental implant in the healed extraction site of the mandibular left first molar (tooth #19). The black arrow indicates the dental implant positioned within the regenerated alveolar ridge, demonstrating adequate bone support following ridge preservation. Radiographic evaluation showed appropriate implant positioning and satisfactory primary stability.

A 5.0 × 14 mm Straumann dental implant was placed according to the manufacturer's surgical protocol with satisfactory primary stability. A healing abutment was immediately connected to permit transmucosal healing while preserving the peri-implant soft tissue architecture and promoting the development of an appropriate emergence profile for the future implant-supported restoration (Figure 5).

**Figure 5:**
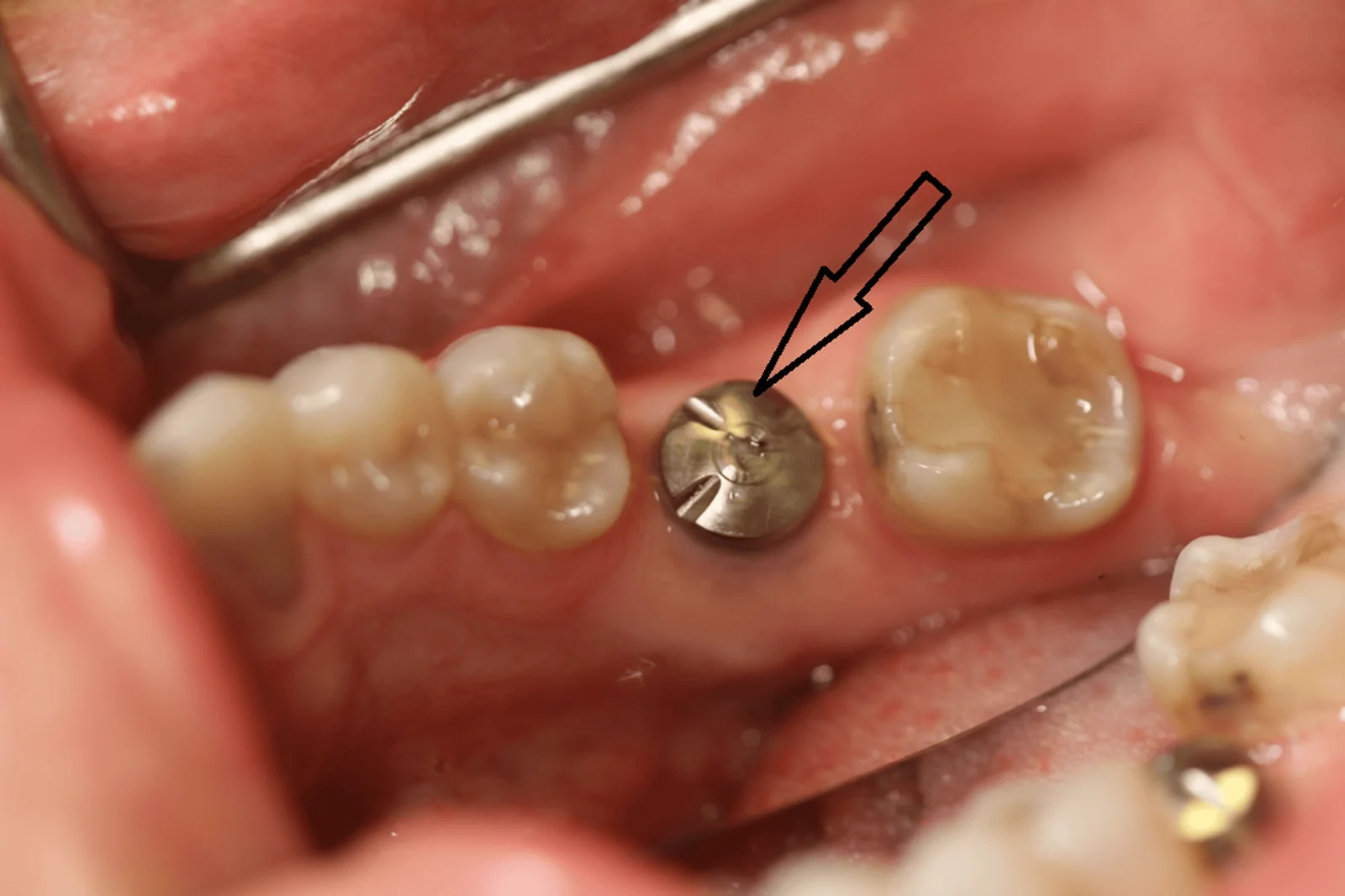
Healing abutment following implant placement. Clinical photograph obtained immediately following placement of a healing abutment onto the dental implant. The black arrow indicates the healing abutment connected to the implant to facilitate transmucosal healing and development of the peri implant soft tissue emergence profile. The healing abutment was placed to preserve the peri implant soft tissue architecture and facilitate fabrication of the definitive implant supported crown.

No intraoperative or postoperative complications were observed. The implant was allowed to heal for an additional three months to facilitate complete osseointegration and maturation of the peri-implant soft tissues. Throughout the healing period, the implant remained clinically stable, with no evidence of pain, mobility, peri-implant inflammation, or infection.

After successful osseointegration was confirmed clinically and radiographically, the restorative phase was initiated. A scan body was connected to the implant, and a digital intraoral scan was obtained to accurately record the implant position for fabrication of the definitive implant-supported restoration using a CAD/CAM workflow. The definitive implant-supported crown was subsequently fabricated and delivered. A postoperative periapical radiograph obtained after crown delivery demonstrated appropriate implant positioning, intimate bone-to-implant contact, stable crestal bone levels, and the absence of peri-implant radiolucency, consistent with successful implant rehabilitation (Figure 6).

**Figure 6:**
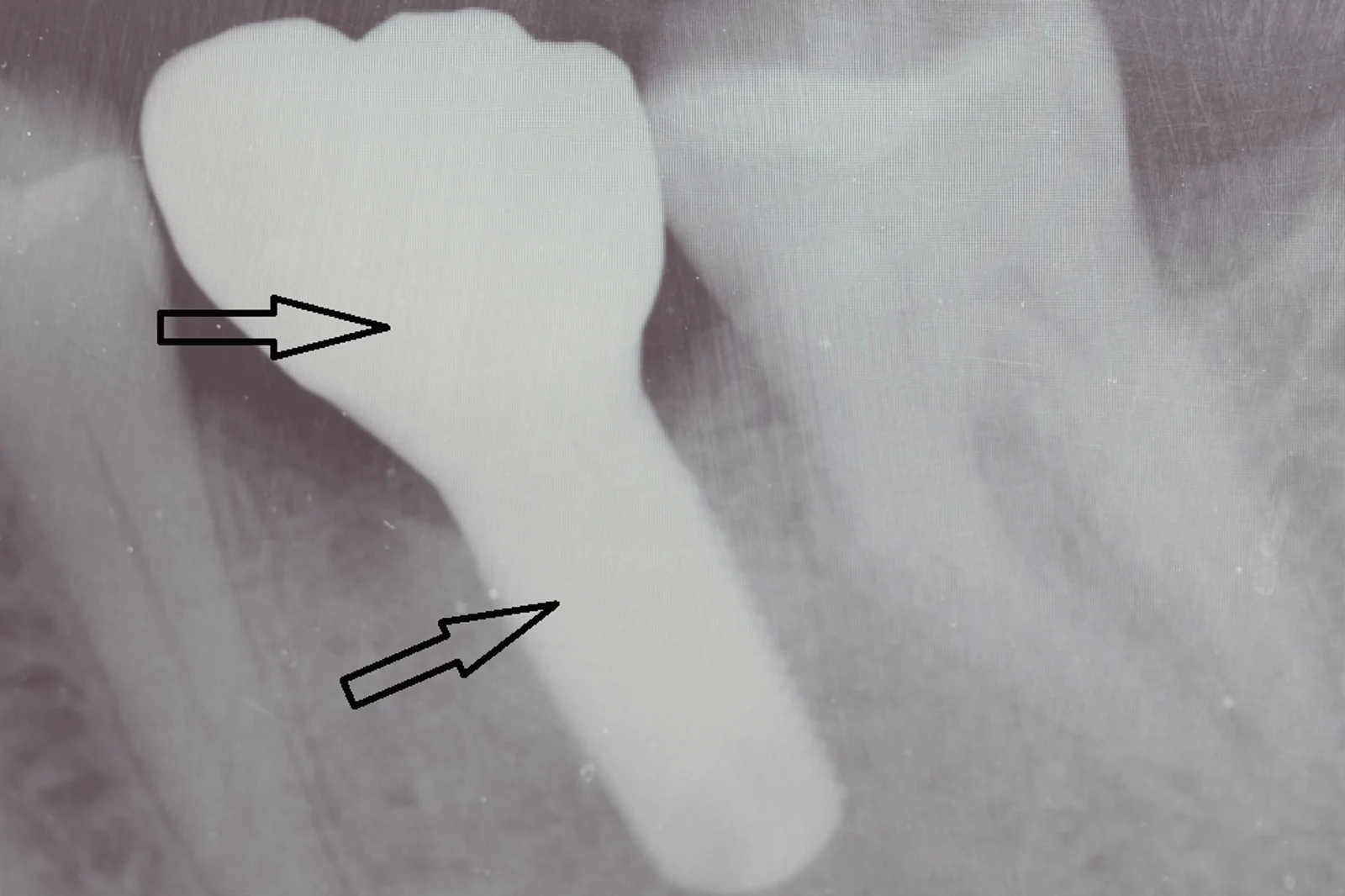
Final implant-supported restoration. Final periapical radiograph demonstrating the definitive implant-supported crown following successful osseointegration of the 5.0 × 14 mm Straumann dental implant. The upper black arrow indicates the definitive implant-supported crown, while the lower black arrow identifies the osseointegrated dental implant within the regenerated alveolar ridge. Radiographic evaluation demonstrated appropriate positioning of the definitive restoration, stable crestal bone levels, intimate bone-to-implant contact, and the absence of peri-implant radiolucency, consistent with successful implant rehabilitation.

Clinical evaluation confirmed excellent marginal adaptation, appropriate proximal contacts, satisfactory occlusion, and favorable esthetics.

Final clinical evaluation demonstrated healthy peri-implant soft tissues with a satisfactory emergence profile and harmonious integration of the restoration with the adjacent dentition. An occlusal clinical photograph demonstrated appropriate occlusal morphology and restoration contours (Figure 7).

**Figure 7:**
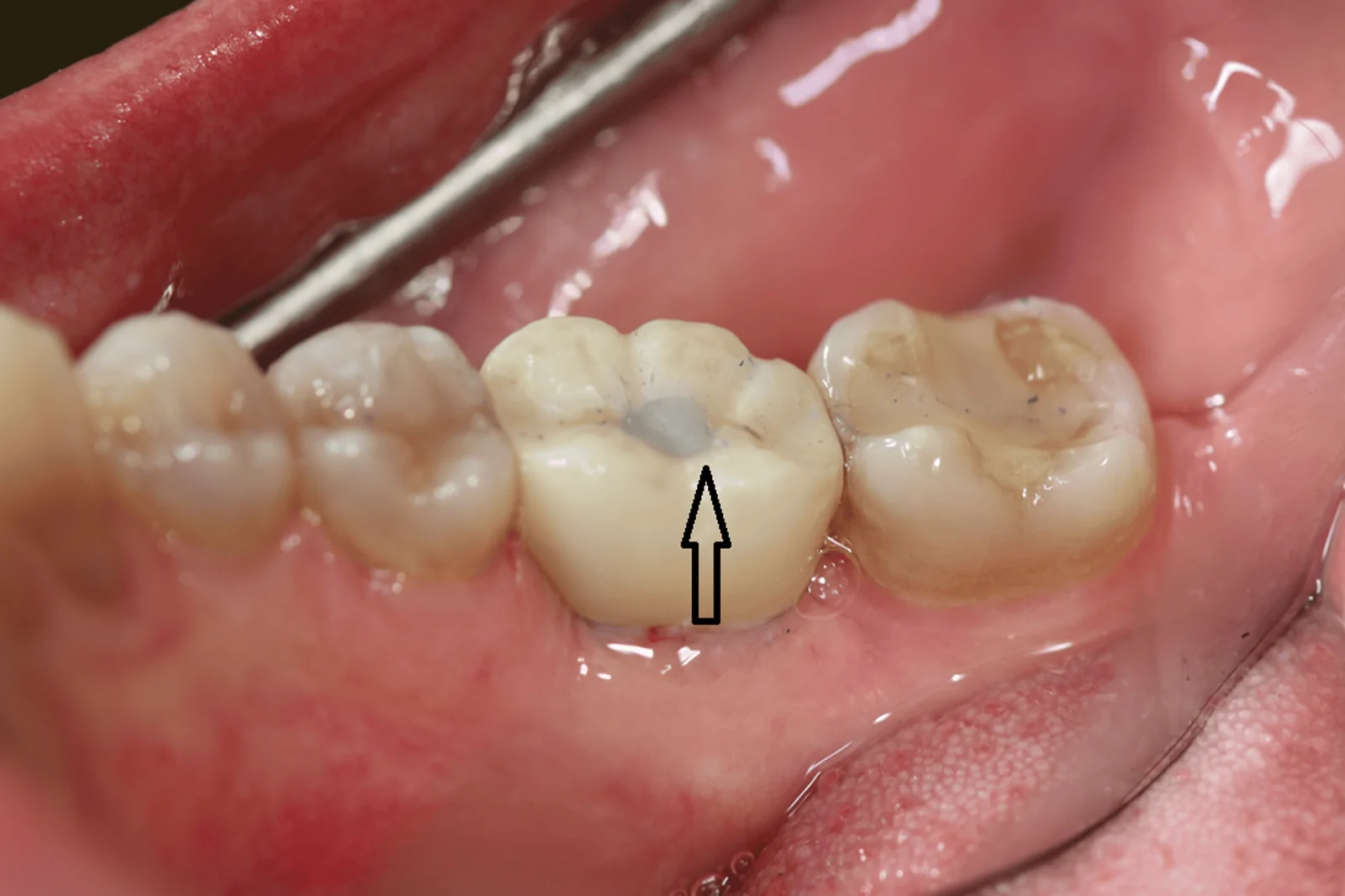
Occlusal view of the definitive implant-supported crown. Occlusal clinical photograph of the definitive implant-supported crown replacing the mandibular left first molar (tooth #19). The black arrow indicates the definitive implant-supported crown. The restoration demonstrates satisfactory occlusal morphology, harmonious integration with the adjacent dentition, appropriate proximal contacts, and healthy peri-implant soft tissues.

The buccal clinical view confirmed excellent marginal adaptation, a well-contoured emergence profile, and healthy peri-implant soft tissues (Figure 8).

**Figure 8:**
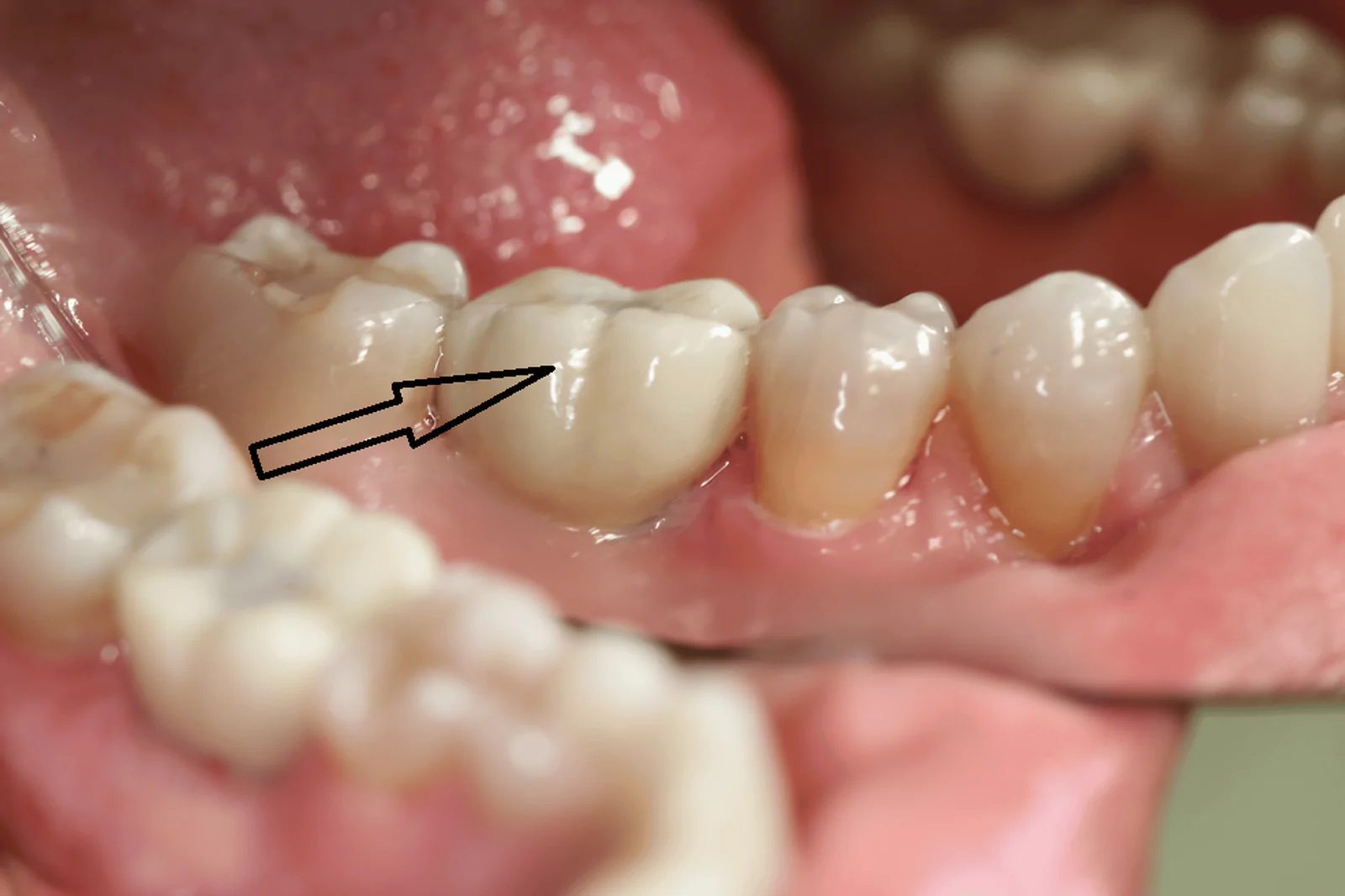
Buccal view of the definitive implant-supported crown. Buccal clinical photograph of the definitive implant-supported crown replacing the mandibular left first molar (tooth #19). The black arrow indicates the definitive implant supported crown. The restoration demonstrates satisfactory marginal adaptation, an appropriate emergence profile, harmonious integration with the adjacent dentition, healthy peri implant soft tissues, and a favorable esthetic contour.

The final documented evaluation was performed at the time of definitive crown delivery, approximately three months after implant placement. Clinical examination demonstrated healthy peri-implant soft tissues, satisfactory occlusion, appropriate marginal adaptation, and favorable esthetic and functional outcomes. The patient reported complete resolution of symptoms and satisfaction with the treatment outcome. No additional follow-up visits were available because the patient did not return for routine maintenance after completion of treatment.

## Discussion

Failure of endodontic treatment remains one of the most common reasons for tooth extraction in adult patients despite the high overall success rates reported for modern root canal therapy. Persistent apical periodontitis, residual intracanal infection, coronal leakage, procedural errors, vertical root fracture, and complex root canal anatomy are among the principal factors associated with treatment failure. When a tooth exhibits an unfavorable long-term prognosis and predictable retreatment is not feasible, extraction followed by implant-supported rehabilitation provides a reliable treatment alternative for restoring oral function and maintaining patient quality of life [1,2].

One of the major challenges following tooth extraction is the physiologic remodeling of the alveolar ridge. Dimensional changes occur rapidly during the first several months of healing and may compromise future implant placement by reducing ridge width and height. Numerous clinical investigations have demonstrated that ridge preservation procedures significantly reduce post-extraction bone loss compared with spontaneous healing, thereby improving the predictability of delayed implant placement and decreasing the need for additional augmentation procedures [3-5].

In the present case, a 68-year-old male patient underwent immediate ridge preservation following extraction of a failed endodontically treated mandibular first molar using DBBM (techBiomat bone®) covered with a resorbable collagen membrane. DBBM has been extensively investigated because of its excellent osteoconductive properties, slow resorption rate, and ability to maintain alveolar ridge dimensions during healing. The material serves as a scaffold for new bone formation while preserving the volume of the extraction socket, making it one of the most widely used grafting materials in implant dentistry [6-8].

The placement of a resorbable collagen membrane further contributed to guided bone regeneration by stabilizing the graft material and preventing migration of epithelial and connective tissue cells into the healing socket. This creates a favorable environment for bone regeneration while eliminating the need for a second surgical procedure for membrane removal. Numerous systematic reviews have reported improved hard tissue preservation when collagen membranes are used in conjunction with particulate bone grafts during ridge preservation procedures [9,10].

Delayed implant placement, approximately three months after ridge preservation, allowed sufficient bone maturation before implant insertion. This treatment protocol has demonstrated high survival rates and predictable osseointegration while reducing the risk of placing implants into immature or inadequately mineralized bone. In this 68-year-old male patient, clinical and radiographic evaluation confirmed satisfactory bone regeneration that permitted placement of a 5.0 × 14 mm Straumann dental implant with excellent primary stability. Following an additional three-month healing period, successful osseointegration was achieved without biological or mechanical complications [11-14].

Implant surface characteristics and surgical protocol are recognized as important determinants of successful osseointegration. Straumann implants have consistently demonstrated excellent long-term survival and stable peri-implant bone levels in both partially and completely edentulous patients. The favorable clinical outcome observed in this case is consistent with previously published evidence supporting the long-term predictability of modern implant systems when appropriate surgical and restorative protocols are followed [15-18].

The restorative phase was completed using a fully digital workflow involving intraoral scanning with a scan body for fabrication of the definitive implant-supported crown. Digital implant impressions have become increasingly popular because they eliminate many limitations associated with conventional impression techniques while improving patient comfort, reducing chair time, and providing accurate transfer of implant position to the dental laboratory. Recent studies have demonstrated that digital workflows produce restorations with excellent marginal adaptation and clinically acceptable accuracy for single implant restorations [19].

The patient achieved satisfactory esthetic and functional outcomes, with healthy peri-implant soft tissues, stable crestal bone levels, appropriate occlusion, and complete resolution of symptoms. The sequential clinical photographs and radiographic documentation demonstrated successful healing throughout each stage of treatment, from extraction and ridge preservation to implant placement and definitive prosthetic rehabilitation.

Although this report describes the successful management of a single patient, it is subject to the inherent limitations of case reports, including the absence of a control group, limited follow-up, and inability to generalize the findings to all clinical situations. Nevertheless, the comprehensive documentation of each treatment stage provides valuable educational information and illustrates a predictable clinical protocol for managing failed endodontically treated teeth requiring implant-supported rehabilitation.

This case highlights the importance of careful treatment planning, preservation of alveolar ridge architecture, appropriate selection of regenerative materials, and the integration of digital restorative techniques to achieve predictable implant outcomes. When supported by proper case selection, healthy peri-implant soft tissue management, and meticulous surgical execution, delayed implant placement following ridge preservation represents a reliable treatment strategy for restoring function and esthetics after extraction of teeth with an unfavorable prognosis [20].

## Conclusions

Delayed implant placement following extraction of a failed endodontically treated mandibular first molar in a 68-year-old male patient, combined with ridge preservation using DBBM and a resorbable collagen membrane, resulted in satisfactory bone regeneration, successful osseointegration, and favorable esthetic and functional outcomes in this patient. The staged treatment approach, combined with a fully digital restorative workflow, facilitated the accurate fabrication of the definitive implant-supported crown and contributed to successful rehabilitation.

This case illustrates that careful treatment planning, alveolar ridge preservation, and evidence-based implant protocols may represent an effective treatment approach for replacing teeth with an unfavorable prognosis in appropriately selected patients. Further studies with larger sample sizes and longer follow-up are needed to confirm these findings.
